# Histopathological Characteristics of Cutaneous Melanoma in Isfahan, Iran, from 2013 to 2018

**DOI:** 10.1155/2022/4490648

**Published:** 2022-09-19

**Authors:** Zahra Malakoutikhah, Fatemeh Mohaghegh, Siavash Karimi, Parvin Rajabi, Elham T. Tabatabaei

**Affiliations:** ^1^Applied Physiology Research Center, Cardiovascular Research Institute, Isfahan University of Medical Sciences, Isfahan, Iran; ^2^Department of Dermatology, Skin Diseases and Leishmaniasis Research Center, School of Medicine, Isfahan University of Medical Sciences, Isfahan, Iran; ^3^School of Medicine, Isfahan University of Medical Sciences, Isfahan, Iran; ^4^Department of Pathology, School of Medicine, Isfahan University of Medical Sciences, Isfahan, Iran

## Abstract

**Objectives:**

To investigate the histopathological characteristics of cutaneous melanoma in Isfahan from 2013 to 2018, according to histopathological subtype, lesions location, Clark level, and Breslow thickness.

**Methods:**

A descriptive, retrospective study in reports of Alzahra Hospital and Dr. Rajabi Pathology Laboratory in Isfahan.

**Results:**

In total, 45 patients were included in this study. The most prevalent histopathological subtype was acral lentiginous melanoma (48.89%), followed by lentigo maligna melanoma (17.78%), nodular melanoma (11.11%), and superficial spreading melanoma (8.89%). Most malignant lesions were on the foot and toes (31.1%) and face (24.4%). Tumor invasion level was mainly at Clark level IV (42.2%). Furthermore, the mean depth of tumor penetration (Breslow thickness) was 3.87 ± 3.35.

**Conclusions:**

Our study revealed the characteristics of melanoma in the Iranian population. Our results showed a similar trend with previous studies in the Asian population. Further investigations are needed to elucidate the role of ethnic and environmental risk factors for developing melanoma in different populations.

## 1. Introduction

Although cutaneous melanoma accounts for a small proportion of all skin cancers diagnosed each year, it is one of the most malignant forms of skin cancer, representing up to 80% of all deaths from skin cancers. Cutaneous melanoma (CM) is also one of the few cancers that are on the rise globally. It is estimated that melanoma incidence has doubled in the last 20 years, becoming a significant health concern worldwide [1].

CM originates from genetically altered and activated melanocytes in the basal epidermis that are producing melanin, the material that protects us against radiation exposure and DNA alteration.

CM is a multifactorial disease, having a heterogeneous presentation. The goal of current strategies is to reduce the disease burden by improving the understanding of the causal factors and their relationships [2].

Cutaneous melanoma varies significantly in genomic profile, clinical manifestations, incidence, and mortality depending on ethnicity, country of residence, degree of sun exposure, and socioeconomic status. Thus, the characteristics of cutaneous melanoma show apparent differences among various countries and nationalities [3]. Moreover, the development of different subtypes of melanoma is not explained by a single evolutionary pathway.

Melanoma's clinical presentation and dermatoscopic features depend on the histopathological type of cancer and the anatomic location of lesions [4]. There are four major types of cutaneous melanoma, including superficial spreading melanoma (SSM), nodular melanoma (NM), lentigo maligna melanoma (LMM), and acral lentiginous melanoma (ALM). Superficial spreading melanoma is the most common histologic subtype in Caucasian populations. This subtype is the most likely to be related to a pre-existing nevus. Furthermore, SSM can appear in any anatomic site, but it is more prevalent in men's back and women's lower extremities [5]. Nodular melanoma is the second most prevalent subtype, which frequently presents a uniform color or amelanotic hue, symmetric borders, and relatively small diameters. These benignant features make timely diagnosis difficult, and most NMs are thicker than 2 mm at the time of diagnosis [6]. Lentigo maligna melanoma is the most prevalent subtype of melanoma on the face, which often occurs in chronically sun-damaged skin of the face and neck of elderly individuals. This subtype's precursor is lentigo maligna (LM) or LMM in situ, which grows slowly for years and eventually progresses to LMM [7]. Although acral lentiginous melanoma is the least prevalent subtype among the Caucasian population, it is the most prevalent subtype in the Asian and dark-skinned population. This subtype is also found in glabrous skin, mainly palms, soles, and subungual areas [8].

Dermatoscopical characteristics of melanomas can indicate their melanocytic origin, including aggregated brown or black globules, pigment networks, and location-related feathers. However, a melanoma may have no clinically and dermatoscopically specific and well-defined features. Indeed there is no exclusive or typical clinical presentation of melanoma. Hence, diagnosis of CM continues to be complicated and challenging for specialists.

The present study aimed to evaluate patients' histopathological features with cutaneous melanoma attending a referral hospital in Isfahan from 2013 to 2018. Hopefully, these findings will help physicians to diagnose melanoma in this area in a more timely and accurate manner.

## 2. Materials and Methods

It was a retrospective, descriptive study of histopathological types of CM in Isfahan, from 2013 to 2018, from the records of Alzahra Hospital and Dr. Rajabi Pathology Laboratory.

Slides with a report of cutaneous melanoma were obtained and re-evaluated by a dermatopathologist. Eligible samples were those that had the patient's data and the patient's pathology sheet. Moreover, the histopathology type, Breslow, and Clark must be visible in the studied slides. If, according to the examining dermatopathologist opinion, the diagnosis of CM was not definitive, the sample would be excluded from the study. If the anonymous review was disagreed with the original report, a secondary anonymous review by another dermatopathologist was considered as a diagnostic criterion.

All samples were reviewed anonymously, without the dermatopathologist knowing the first diagnosis of each slide. Histopathological type of cutaneous melanoma, Clark's invasion level, Breslow thickness, ulcer status, and vascular invasion were determined for each sample and specified in a relevant table. Demographic data, including the patient's age, gender, and address, were also registered.

All data were uploaded in SPSS Ver 16 software. Statistical analysis was performed using the independent *t*-test and chi-square test (Fisher's exact test) for categorical values comparison (*P*-value <0.05).

## 3. Results

**Figure 1:**
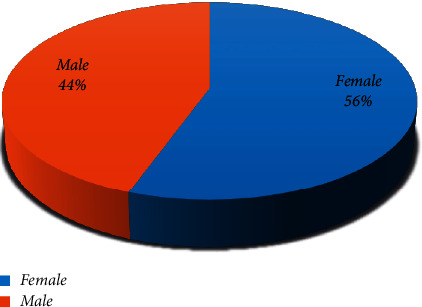
Distribution of cutaneous melanoma according to gender.

Forty-five patients with a confirmed diagnosis of CM were found, of which 44.4% (20 cases) were male, and 55.6% (25 cases) were female with a male to female ratio of 0.8 : 1 [Figure 1].

The mean age of all patients was 63.31 ± 15.38, with a range of 32 to 92 years. The age variable follows the normal distribution. The oldest group was nodular melanoma patients [Table 1].

**Figure 2:**
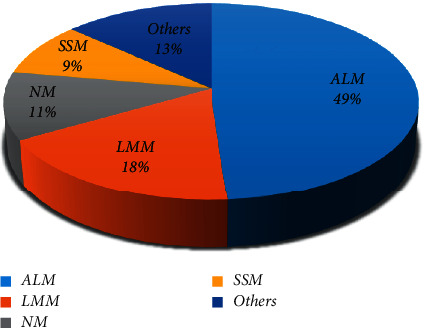
Distribution of cutaneous melanoma according to histopathological subtype.

According to the frequency of different histopathologic types, the most common type seen in our study was ALM (48.89%), followed by LLM type (17.78%). Moreover, histopathologic types' frequency had no significant differences between men and women (*P*=0.97) and between different age groups (*P*=0.86) [Figure 2].

The mean depth of tumor penetration (Breslow thickness) was 3.87 ± 3.35 mm with a range of 0.8–15 mm in the present study. The tumor penetration depth does not follow the normal distribution [Table 2].

The highest rate of tumor invasion was to the reticular dermis (Clark IV) and then to the fat layer (Clark V), 42.2% and 22.2%, respectively [Table 2]. The most common tumor lesion site was the foot and toes (31.1%), followed by the face (24.4%). The lesion site's frequency distribution based on age, sex, and Breslow thickness is summarized in Table 3.

Vascular invasion was seen in eight patients (17.8%), of which 62.5% were seen in ALM type. Furthermore, 19 patients (42.2%) had ulcers. The highest ulcer rate was in the ALM type (52.63%) and then in NM and LMM types (15.78%). The frequency distribution of vascular invasion and ulceration in the samples based on the depth of tumor penetration and the lesion's location is shown in Table 4.

## 4. Discussion

The increasing prevalence of cutaneous malignant melanoma in recent years has attracted the attention of many researchers. Our study revealed the histopathological characteristics of melanoma in patients referred to Alzahra Hospital and Dr. Rajabi Pathology Laboratory in Isfahan, from 2013 to 2018. Our melanoma patients' mean age was 63.31 ± 15.38, with a range of 32–92 years. These findings show the same trend as previous studies. Notably, the most prevalent histopathological subtype was ALM (48.89%), followed by LMM (17.78%), NM (11.11%), and SSM (8.89%) [Figure 2]. As previous studies conducted in Iran's different cities showed, the most common histopathological subtype in our study was ALM. The prevalence of the ALM subtype in the Kamyab et al.'s study [9] in Tehran was 30%, and in the Handjani et al.'s study [10] in southern Iran was 44.8%. These results are consistent with other research studies conducted in the Asian and dark-skinned populations [11, 12].

According to the SEER database, ALM was predominant among African–Americans, while the most common histopathologic subtype in other racial and ethnic groups in the United States was SSM [12, 13]. A large-scale study conducted in the Japanese population reported ALM as the most prevalent subtype (40.4%). In contrast, the proportion of this subtype in non-Hispanic whites was only 1.5% [14]. Based on these and other valid studies, we can conclude that the ALM is the most prevalent histopathological subtype in Asian and dark-skinned populations. Thus, geographical and racial variations in melanoma's clinical manifestations have been confirmed. The high prevalence of ALM might be a significant cause of the high mortality rate among these races despite the low incidence rate [3, 15]. According to the SEER study, African–Americans had significantly shorter melanoma-specific survival than Caucasians. In African–Americans melanoma patients with NM or ALM subtype, melanoma-specific mortality was statically higher, while 5-year survival was statically lower. Interestingly, African–Americans experienced higher melanoma-specific mortality and lower survival for NM and ALM than Caucasians [12].

In several other studies, the survival of the ALM subtype has also been compared with other subtypes. According to a study conducted by Ishihara et al., the survival of ALM was significantly lower than SSM in the Japanese population. Fujisawa et al. reported no association between ALM and survival across the whole study. However, they found a significant survival disadvantage in the ALM stage IIIA. It can be understood that ALM worsens the prognosis of melanoma in the early stages of lymphatic spread, and this type may need to be evaluated for the indication of additional treatments such as SLNB and adjuvant therapy [14, 16].

ALM has also been reported as an independent negative predictor of melanoma-specific survival in different studies [12, 16, 17]. An extensive nationwide study in the Netherlands showed that NM and ALM subtypes had lower survival than SSM and LMM. In Gumaste et al.'s study, the hazard ratio of survival for ALMs vs. non-ALMs was 2.64 (*P*=0.001). Moreover, the ALM subtype's recurrence rate was statically more than the others (49% vs. 30%, *p*=0.007), particularly in tumors with less than 2 mm penetration depth. These pieces of evidence suggest that ALM is a tough variant of melanoma and may require unique and more invasive treatments [16, 17].

**Figure 3:**
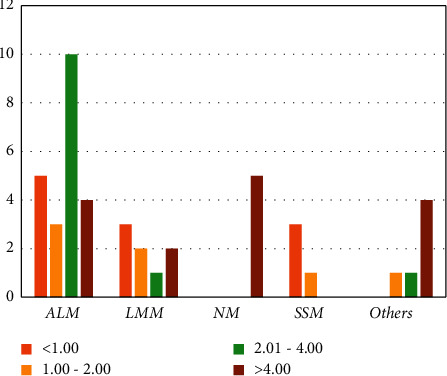
Breslow thickness in different types of melanoma.

**Figure 4:**
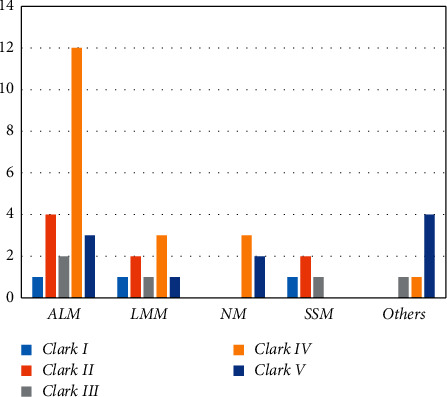
Clark's invasion level in different types of melanoma.

Various possible explanations have been put forward for these pieces of evidence. Patients with ALM often have deeper, more advanced stages, and a higher rate of ulceration at the time of diagnosis. Tumor thickness and ulceration have been reported as the most influential independent predictors of survival for melanoma [13, 18]. In our study, most ALM cases (45.5%) had Breslow thicknesses of 2.1 to 4 mm [Figure 3, Table 1], and 54.5% were diagnosed at Clark invasion level IV [Figure 4, Table 1].

The most common Breslow thickness across the entire study was more than 4 mm (35.6%), and 42.2% of melanoma patients were diagnosed at Clark invasion level IV [Table 2]. According to Lee et al.'s study, the mean Breslow thickness in ALM subtypes was 2.5 ± 2.3 mm, while this amount in non-ALM subtypes was only 1.7 ± 2.0 mm (*P*=0.30). The highest rate of ulceration in the current study was also seen in the ALM subtype (52.62%) [15].

The anatomic location and atypical presentation of ALM may also contribute to the poorer prognosis of ALM. In our study, the most common tumor sites were foot and toes (31.1%) [Table 3]. In fact, the most common areas where ALM occurs are less likely to be noticed. So we diagnose this subtype late, in advanced stages, with high penetration depth and high rate of ulceration. In Lee et al.'s study, the mean duration of melanoma diagnosis in all groups was 20 months, ranging from 1 to 120 months. In contrast, the mean duration of diagnosing ALM and non-ALM was 27 ± 33 and 12 ± 14 months, respectively. Notably, when this variable was stratified against ethnicity, the delay was reported to be 22 ± 28 months in Asians and 7 ± 5 months for Caucasians (*P*=0.09) [15].

However, in various studies, the ALM subtype's survival rate remained lower than other subtypes, even after controlling melanoma stages. Evidence exists to show that inherent biologic differences in melanoma subtypes may also play an essential role in the prognosis and even therapeutic strategy of cutaneous melanoma. Patients with ALM have been shown to demonstrate specific genetic aberrations, indicating that the potency of invasion and ALM progression may be biologically distinct from other types of cutaneous melanoma [18]. Moreover, in contrast to Caucasians, the lower incidence rate and less awareness about melanoma in dark-skinned and Asian populations lead to a delay in diagnosis and advanced stages at the presentation. Socioeconomic status is also an important prognostic factor among these groups. This evidence highlights the importance of raising awareness about melanoma for all racial and ethnic groups, particularly Asians [12].

Our study's primary limitation to the generalization of these results is the small sample size. However, our results showed the same trend as other studies.

## 5. Conclusion

Our study showed that the ALM is the most prevalent melanoma subtype among the Iranian population. Our findings regarding the histopathology subtype, anatomic location, tumor thickness, and Clark invasion level were also in agreement with other studies conducted in the Asian population.

## Figures and Tables

**Table 1:** The frequency distribution of multiple variables based on histopathologic subtypes of cutaneous melanoma.

Histopathologic types of cutaneous melanoma					
	ALM	LMM	NM	SSM	Others
Number	22	8	5	4	6
Percent	48.89	17.78	11.11	8.89	13.33

Gender (%)					
Male	10 (45.5)	4 (50)	2 (40)	2 (50)	2 (33.3)
Female	12 (54.5)	4 (50)	3 (60)	2 (50)	4 (66.7)
Mean age ± SD	62.00 ± 15.25	69.13 ± 15.18	70.20 ± 15.02	60.25 ± 8.88	56.67 ± 19.61

Clark's invasion level (%)					
Level I	1 (4.5)	1 (12.5)	—	1 (25.0)	—
Level II	4 (18.2)	2 (25.0)	—	2 (50.0)	—
Level III	2 (9.1)	1 (12.5)	—	1 (25.0)	1 (16.7)
Level IV	12 (54.5)	3 (37.5)	3 (60.0)	—	1 (16.7)
Level V	3 (13.6)	1 (12.5)	2 (40.0)	—	4 (66.7)

Depth of tumor penetration (Breslow thickness) (%)					
<1.00	5 (22.7)	3 (37.5)	—	3 (75.0)	—
1.00–2.00	3 (13.6)	2 (25.0)	—	1 (25.0)	1 (16.7)
2.01–4.00	10 (45.5)	1 (12.5)	—	—	—
>4.00	4 (18.2)	2 (25.0)	5 (100)	—	5 (83.3)
Mean ± SD	3.11 ± 2.16	2.48 ± 2.15	7.90 ± 4.56	1.10 ± 0.60	7.03 ± 3.99

Tumor location (%)					
Face	—	7 (87.5)	2 (40.0)	—	2 (33.3)
Scalp	—	1 (12.5)	—	—	—
Trunk	—	—	1 (20.0)	2 (50.0)	1 (16.7)
Arm	—	—	—	2 (50.0)	1 (16.7)
Hand	7 (31.8)	—	—	—	—
Anus	—	—	1 (20.0)	—	—
Leg	1 (4.5)	—	1 (20.0)	—	2 (33.3)
Foot	14 (63.6)	—	—	—	—

Moreover, the mean age in men was 65.55 ± 16.29, and in women was 61.52 ± 14.70, which was not statistically significant (*P*=0.38).

**Table 2:** Frequency distribution of Breslow thickness and tumor invasion level.

Variable	Number	Percent
Depth of tumor penetration (Breslow thickness)		
<1.00	11	24.4
1.00–2.00	7	15.6
2.01–4.00	11	24.4
>4.00	16	35.6
Total	45	100

Clark's invasion level		
Level I: limited to the epidermis	3	6.7
Level II: invasion of the papillary dermis	8	17.8
Level III: filling the papillary dermis to connect the reticular dermis	5	11.1
Level IV: invasion of the reticular dermis	19	42.2
Level V: fat invasion	10	22.2
Total	45	100

**Table 3:** The frequency distribution of lesion site based on sex, age, and Breslow thickness.

Tumor location	Number	Percent	Gender (%)		Mean age ± SD	Breslow's thickness
			Male	Female		
Face	11	24.4	6 (30.0)	5 (20.0)	69.55 ± 16.80	3.35 ± 2.47
Scalp	1	2.2	—	1 (4.0)	76.00	6.00
Trunk	4	8.9	3 (15.0)	1 (4.0)	63.50 ± 7.32	6.15 ± 6.29
Arm	3	6.7	—	3 (12.0)	59.67 ± 6.65	3.93 ± 4.42
Hand	7	15.6	—	7 (28.0)	59.57 ± 13.37	3.10 ± 1.27
Anus	1	2.2	—	1 (4.0)	73.00	15.00
Leg	4	8.9	2 (10.0)	2 (8.0)	51.25 ± 19.36	4.17 ± 2.07
Foot	14	31.1	9 (45.0)	5 (20.0)	62.86 ± 16.94	2.98 ± 2.54
Total	45	100	20 (100)	25 (100)		

**Table 4:** The frequency distribution of vascular invasion and ulceration in the samples based on the lesion's location and depth of tumor penetration.

Variable	Vascular invasion (%)		Ulceration (%)	
	Yes	No	Yes	No
The whole sample	8 (17.8)	37 (82.2)	19 (42.2)	26 (57.8)

Tumor location				
Face	1 (12.5)	10 (27.0)	6 (31.6)	5 (19.2)
Scalp	—	1 (2.7)	—	1 (3.8)
Trunk	1 (12.5)	3 (8.1)	1 (5.3)	3 (11.5)
Arm	1 (12.5)	2 (5.4)	1 (5.3)	2 (7.7)
Hand	1 (12.5)	6 (16.2)	5 (26.3)	2 (7.7)
Anus	—	1 (2.7)	1 (5.3)	—
Leg	1 (12.5)	3 (8.1)	1 (5.3)	3 (11.5)
Foot	3 (37.5)	11 (29.7)	4 (21.1)	10 (38.5)
Total	8 (100)	37 (100)	19 (100)	26 (100)

Depth of tumor penetration (Breslow thickness)				
<1.00	—	11 (29.7)	1 (5.3)	10 (38.5)
1.00–2.00	—	7 (18.9)	3 (15.8)	4 (15.4)
2.01–4.00	1 (12.5)	10 (27.0)	6 (31.6)	5 (19.2)
>4.00	7 (87.5)	9 (24.3)	9 (47.4)	7 (26.9)
Total	8 (100)	37 (100)	19 (100)	26 (100)
Mean ± SD	7.12 ± 3.18	3.17 ± 2.98	5.08 ± 3.51	2.99 ± 2.98

## Data Availability

All data that support the findings of this study and the patient's consent are openly available from Dr. Fatemeh Mohaghegh, the corresponding author.
